# Abundance and Infestation of Mites on Bower’s White-Toothed Rat (*Berylmys bowersi*) in Southwest China

**DOI:** 10.3390/vetsci12050426

**Published:** 2025-04-30

**Authors:** Chenxi Liu, Xianguo Guo, Yan Lv, Pengwu Yin, Wenyu Song, Peiying Peng, Rong Xiang, Yanling Chen, Bei Li

**Affiliations:** 1 Institute of Pathogens and Vectors, Yunnan Provincial Key Laboratory for Zoonosis Control and Prevention, Dali University, Dali 671000, China; oolxcoo@163.com (C.L.); lv13987290703@163.com (Y.L.); pengwuyin@vip.163.com (P.Y.); merlin_song@hotmail.com (W.S.); xiangrong102501@163.com (R.X.); lb909150@163.com (B.L.); 2 Institute of Microbiology, Qujing Medical College, Qujing 655100, China; peiyingpeng@hotmail.com; 3 Shenshan Central Hospital, Sun Yat-Sen Memorial Hospital, Sun Yat-Sen University, Shanwei 516600, China; lynne0327@163.com

**Keywords:** chigger mite, gamasid mite, infestation, rodent, southwest China

## Abstract

Chiggers (chigger mites) and gamasid mites are two groups of ectoparasites on rodents, and they can be the vectors or potential vectors of scrub typhus and hemorrhagic fever with renal syndrome (HFRS), while Bower’s white-toothed rat (*Berylmys bowersi*) can serve as the reservoir host of the diseases. Based on field investigations at 117 survey sites of southwest China, a total of 2512 mites were collected from 55 *B. bowersi* rats. Under a microscope, these mites were taxonomically identified as 56 distinct species, comprising 37 chigger mite species and 19 gamasid mite species. The mite infestation burdens on *B. bowersi* were heavy, with a high prevalence (*P_M_* = 85.45%), mean abundance (*MA* = 45.67), and intensity (*MI* = 53.45). Of 56 mite species identified, 7 are vectors of scrub typhus and HFRS. Of the seven vector mite species, *Leptotrombidium scutellare* was one of dominant chigger species, with a higher infestation index on rats (*P_M_* = 21.82%, *MA* = 7.76) than the other six vector mite species. The sex ratio of female gamasid mites was higher than that of males. The number and infestation of adult gamasid mites were higher than those of immature mites. The infestation indexes of mites on *B. bowersi* hosts varied with the host’s sex and age and fluctuated along different environmental gradients. The association coefficient (*V*) showed a slight positive association between chiggers and gamasid mites, suggesting that these two groups of mites may coexist on *B. bowersi* rats. Spearman correlation coefficients showed positive correlations among some dominant and vector mite species, indicating that some mite species tend to select the same hosts. A total of 69 mite species (47 chigger mite species and 22 gamasid mite species) on *B. bowersi* were estimated by species rarefaction and extrapolation curves. In conclusion, *Berylmys bowersi* has a high susceptibility to mite infestation, and it has the potential to harbor abundant mites, with heavy mite burdens. The occurrence of vector mite species, especially *L. scutellare* (the dominant species), increases the potential risk of transmission and the focus persistence of scrub typhus and HFRS in southwest China.

## Simple Summary

Field surveys were carried out at 117 sites in southwest China. Based on the taxonomic identification under a microscope, 2512 mites collected from 55 Bower’s white-toothed rats (*Berylmys bowersi*) were identified as 56 species (37 chigger mites and 19 gamasid mites). The mite infestation burdens on rats were heavy, with a high prevalence (*P_M_* = 85.45%) and intensity (*MA* = 45.67 and *MI* = 53.45). Of 56 mite species, 7 were vectors of zoonotic diseases. The vector chigger mite *L. scutellare* had a higher infestation on rats (*P_M_* = 21.82%, *MA* = 7.76) than the other six vector mite species. Most gamasid mites had more females than males and more adults than immature mites. The mite infestation was different on different sexes and ages of rat hosts and fluctuated in different environments. Chigger mites and gamasid mites had a tendency to occur on rat hosts simultaneously, with a slight positive association.

## 1. Introduction

Rodents (rats, mice, voles, etc.) are not only famous pests in agriculture and forestry but also the important infectious source and reservoir host of many zoonoses (zoonotic diseases) such as plague, scrub typhus (tsutsugamushi disease), murine typhus, and hemorrhagic fever with renal syndrome (HFRS) [1,2,3,4]. Rodents often harbor ectoparasites on their body surface, and chiggers (chigger mites) and gamasid mites are common groups of ectoparasites associated with rodents [5,6,7]. Chiggers are the exclusive vector of scrub typhus and they can also be the potential vector of HFRS [8,9,10,11], and gamasid mites can serve as the vector or potential vector of rickettsial pox, HFRS, and some other zoonoses [11,12,13]. Through the biting activity of mites, the pathogens of these zoonoses can be transmitted among different animal hosts (rodents and other wild animals) and even from animal hosts to humans [10,11,14]. Scrub typhus and HFRS are two common zoonotic diseases, and the incidence of these diseases has shown an escalating trend in many places with continuously expanding foci in recent years [10,15,16,17,18,19]. Southwest China is an important focus of scrub typhus, HFRS, and other zoonoses [16,20,21,22,23,24], and it is of medical significance to study rodent-associated mites in the region.

Bower’s white-toothed rat, *Berylmys bowersi* (Anderson, 1878), is a giant rat species, which was first named *Mus bowersi* by Anderson in 1878, and eventually accepted with the species name *Berylmys bowersi* after Musser and Newcomb proposed that the species *bowersi* should be placed in the genus *Berylmys* [25]. *Berylmys bowersi* is mainly distributed in tropical and subtropical areas, including Thailand, Malaysia, Myanmar, and southern and southwestern China [25,26,27,28,29]. Besides harming agricultural and forestry plants in distribution areas, *B. bowersi* can also serve as the infectious source and reservoir host of leptospirosis, scrub typhus, and other zoonoses [25,26,30,31,32,33]. In recent years (2019–2023), other pathogens have been successively detected from *B. bowersi* rats, including *Borrelia miyamotoi* (a spirochete causing relapsing fever) and two species of opportunistic pathogenic protozoa, *Enterocytozoon bieneusi* and *Cryptosporidium viatorum* [29,31,33]. Although *B. bowersi* is of medical importance, little research has been carried out on its associated ectoparasites to date. In a long-term field survey conducted in southwest China from 2001 to 2024, we collected and identified a large number of chiggers and gamasid mites on the body surface of *B. bowersi* rats, which piqued our interest in retrospectively analyzing the infestation and related ecological issues of mites on the rat. The present study enriches the understanding of this giant rat species and its associated mites, and it provides scientific information for further studies and the surveillance and control of related mite-borne zoonoses.

## 2. Materials and Methods

### 2.1. Collection and Identification of Mites and Their Rodent Hosts

**Figure 1:**
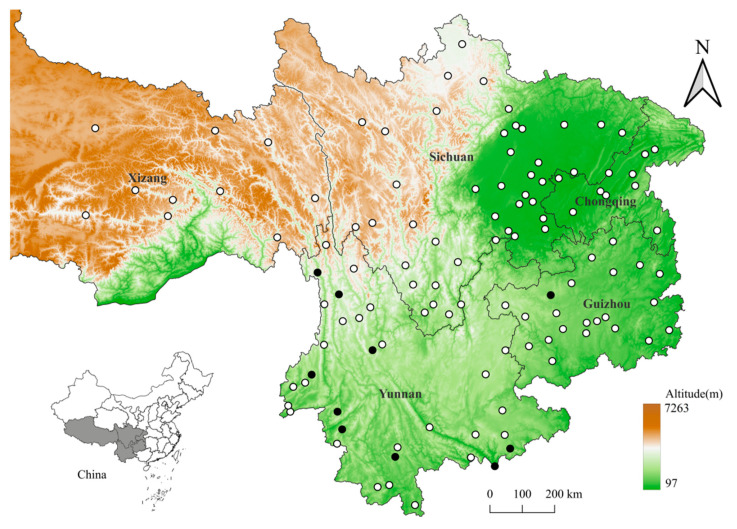
Distribution of 117 survey sites in the five provincial regions of southwest China (2001–2024). Annotation: The hollow circles (○) represent 117 survey sites, and the solid circles (●) represent 10 positive sites where Bower’s white-toothed rats (*Berylmys bowersi*) were captured.

The present study is a retrospective investigation. The original data came from a long-term field investigation at 117 survey sites across the five provincial regions of southwest China (97°21′–110°11′ E, 21°08′–33°41′ N) between 2001 and 2024. The five provincial regions are Yunnan, Guizhou, Sichuan, Chongqing, and Xizang (Tibet). In the vast territory of Xizang, however, only the eastern part was investigated, as the western part of Xizang is a vast and sparsely populated territory with relatively inconvenient transportation, the existence of hypoxia, and potential risks in the sparsely populated, high, cold areas, and in addition, we had insufficient human resources and financial support to cover the whole territory of Xizang (Figure 1 in Section 3). At each survey site, mousetraps (18 × 12 × 9 cm; Guixi Mousetrap Apparatus Factory, Guixi, Jiangxi, China) were placed to capture rodents in different habitats, including indoor habitats (houses, stables, barns, and nearby surroundings) and outdoor habitats (farmlands, bushes, and woodlands). In dry lands, every 25 mousetraps (cage traps) in a group were placed in a straight line, with a spacing of 5 m and a row spacing of 20 m. Considering the complexity and diversity of environmental conditions in actual investigations, the placement of mousetraps was flexibly adjusted according to specific environmental conditions. For example, indoors, a mousetrap was placed every 15 square meters (15 m^2^) along the base of a wall. In a paddy field, mousetraps were placed along the bank of the field. The same number of mousetraps was placed at each survey site to ensure the “homogeneity” and “comparability” of sampling methods at different survey sites. Each rodent host captured was separately placed in a cloth bag and transported to the temporary field laboratory [34,35,36]. In the temporary laboratory, chiggers and gamasid mites on the body surfaces of rodent hosts were collected in the conventional way [37,38]. After the collection of mites, each rodent host was identified into species based on its morphology [39,40,41,42]. The collected mites were mounted with Hoyer’s solution onto glass slides. After the process of dehydration, transparency, and drying, each mite specimen was identified into species under a microscope (Olympus Corporation, Tokyo, Japan) based on the related taxonomic literature and identification keys [43,44,45,46]. After the taxonomic identification of all rodent hosts and mites was completed, Bower’s white-toothed rats (*B. bowersi*) were screened as the subject of this study.

### 2.2. Statistical Analysis

Based on the field investigation and taxonomic identification, the constituent ratio (*C_r_*) was used to calculate the percentages of mites and their rat hosts (*B. bowersi*). The prevalence (*P_M_*), mean abundance (*MA*), and mean intensity (*MI*) were calculated to reflect the percentage of infested hosts with mites, the average number of mites per examined host, and the average number of mites per infested host [37,47,48]. The sex ratio was used to illustrate the constituent ratio of females and males [49]. All the above indices were calculated with Microsoft Excel, the Chi-square test was used for the comparison of *P_M_*, and the non-parametric test was used for *MA* and *MI*. These significance analyses were performed in SPSS 20.0. The Margalef index (*M_f_*), Shannon–Wiener diversity index (*H′*), Pielou evenness (*E*), and Simpson’s dominance index (*D*) were used to analyze the basic characteristics of mite communities [50,51,52], as calculated in R software (version 4.3.3) with the package “vegan”. After calculating the Spearman correlation coefficient (r) using the “cor” function in R software, the “corrplot” package was used to visualize the interspecific relationships among dominant and vector mites. The values of correlation coefficients (r) ranged from −1 to 1 [53,54]. The association coefficient (*V*), which also ranged from −1 to 1, was used to analyze the mutual relationship between two groups of mites: all chiggers and gamasid mites [38,47,50]; it was calculated in SPSS 20.0, and the Chi-square test was used to test the significance of *V*. The estimated species richness was calculated and visualized through the curves of species rarefaction and extrapolation by running the relevant functions in the “INEXT” package in R software [55,56]. The following are formulas related to the above statistics:

(1) $$C_{r}=\frac{N_{i}}{N}\times 100\%$$

(2) $$P_{M}=\frac{H_{m}}{H}\times 100\%$$

(3) $$\mathrm{MA}=\frac{N_{i}}{H}$$

(4) $$\mathrm{MI}=\frac{N_{i}}{H_{m}}$$

(5) $$M_{f}=\frac{(S-1)}{\ln N}$$

(6) $$H^{\prime }=-\sum_{i=1}^{S}\frac{N_{i}}{N}\ln\frac{N_{i}}{N}$$

(7) $$D=1\;-\;\sum_{i=1}^{S}({\frac{N_{i}}{N})}^{2}$$

(8) $$E=\frac{H^{\prime }}{\ln S}$$

(9) $$V=\frac{ad-bc}{\sqrt{\left(a+b\right)\left(c+d\right)\left(b+d\right)\left(a+c\right)}}$$

(10) $$r=1-\frac{6\sum t^{2}}{n(n^{2}-1)}$$

In the above formulas, *N_i_* = the number of a certain mite species (species *i*); *N* = the total number of all mites collected; *H_m_* = the number of animal hosts infested; *H* = the total number of animal hosts examined; *S* = the number of mite species (species richness); *V* = association coefficient between two groups of mites: chigger mites and gamasid mites; *a* = host individuals on which both chigger species and gamasid species concurrently appear; *b* = host individuals on which chigger species appear, but gamasid species do not appear; *c* = host individuals on which gamasid species appear, but chigger species do not appear; *d* = host individuals on which neither chigger species nor gamasid species appear; “*n*” = the number of samples; We assume that species X has the rank (*R_X_*) and species Y has the rank (*R_Y_*), “*t*” = *R_X_* − *R_Y_*.

## 3. Results

### 3.1. Species and Abundance of Mites on Berylmys bowersi

**Figure 2:**
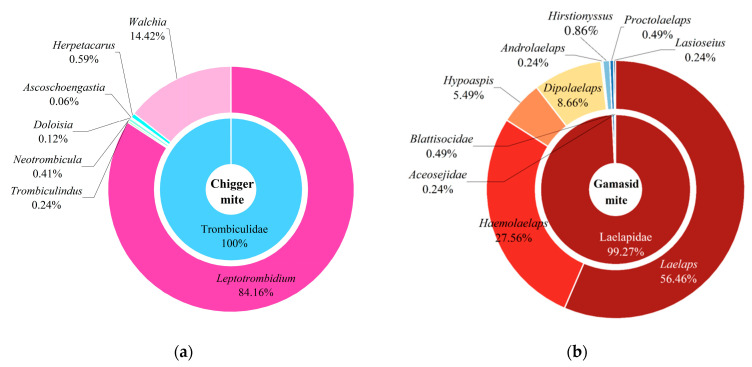
Visualized sunburst charts for the constituent ratios of different families and genera of chiggers (**a**) and gamasid mites (**b**) identified from *B. bowersi* rats in southwestern China (2001–2024).

**Figure 3:**
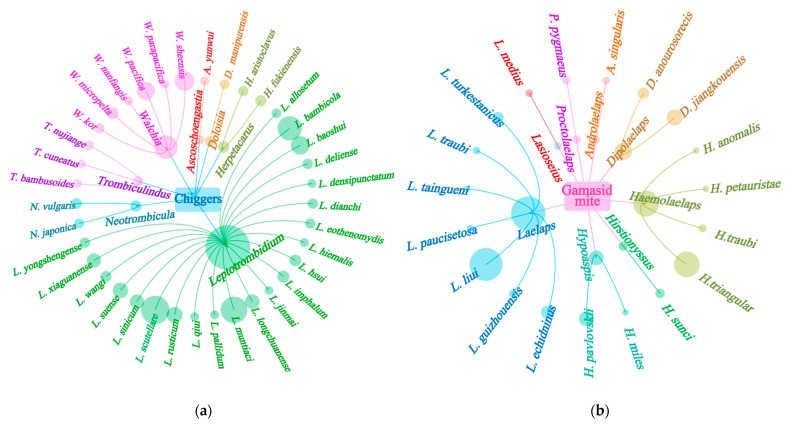
Visualized hierarchical network diagrams for the constituent ratios (*C_r_*) of different chigger species (**a**) and gamasid mite species (**b**) identified from *B. bowersi* rats in southwestern China (2001–2024). Annotation: The circle size represents the constituent ratio (*C_r_*) of corresponding mites. The bigger the circle, the higher the *C_r_*.

A total of 55 Bower’s white-toothed rats (*B. bowersi*) were captured at 10 out of 117 survey sites in southwest China, and 2512 mites (1692 chiggers and 820 gamasid mites) collected from the rats were identified as being from 4 families, 15 genera, and 56 species. Of the identified 56 mite species, chiggers accounted for 1 family (Trombiculidae), 7 genera, and 37 species, and gamasid mites accounted for 3 families (Laelapidae, Blattisocidae and Aceosejidae), 8 genera, and 19 species (Figure 1, Table 1). A sunburst chart was used to visualize the constituent ratios (*C_r_*, %) of different families and genera of two mite groups (chiggers and gamasid mites), and a hierarchical network diagram was used to visualize the constituent ratios of different mite species (Figure 2 and Figure 3). At the genus level, the genus *Leptotrombidium* accounted for 84.16% of the total chiggers (*C_r_* = 84.16%, 1424/1692), and the genus *Laelaps* in the family Laelapidae accounted for 56.46% of the total gamasid mites (*C_r_* = 56.46%, 463/820) (Figure 2). At the species level, there were three dominant chigger species and two dominant gamasid mite species. The three dominant chigger species are *Leptotrombidium scutellare* (*C_r_* = 25.24%, 427/1692), *L. muntiaci* (*C_r_* = 20.98%, 355/1692), and *L. bambicola* (*C_r_* = 15.72%, 266/1692). The two dominant gamasid mites are *Laelaps liui* (*C_r_* = 50.73%, 416/820) and *Haemolaelaps triangular* (*C_r_* = 26.95%, 221/820) (Table 1, Figure 3). Among the 56 mite species found on *B. bowersi*, there were seven vector mite species, including six chigger species and one gamasid mite species, and the total *C_r_* of the seven vector species reached 24.92% of all mites (*C_r_* = 24.92%, 626/2512). The six vector chigger species are *L. deliense*, *L. scutellare*, *L. rusticum*, *L. imphalum*, *L. pallidum,* and *Walchia Pacifica*, which are the vectors of scrub typhus, and *L. scutellare* is also the potential vector of HFRS. The vector gamasid mite species is *L. echidninus*, which can serve as the potential vector of HFRS (Table 1).

### 3.2. Variations in Mite Infestation on B. bowersi

**Figure 4:**
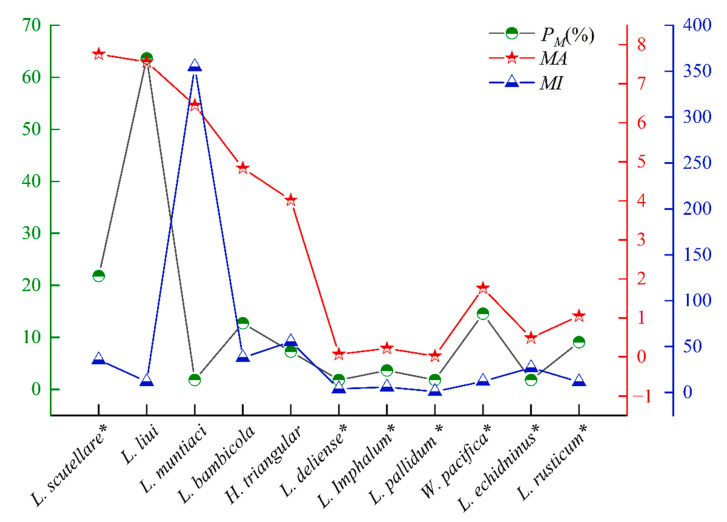
The prevalence (*P_M_*), mean abundance (*MA*), and mean intensity (*MI*) of dominant and vector mites. Annotation: The mark “*” represents the vector and potential vector species of mites. Five dominant mite species: The standard deviation and confidence intervals are 30.81 and 2.74–10.33 for *MA*, and 61.90 and 14.90–47.03 for *MI*. Seven vector mite species: The standard deviation and confidence intervals are 13.09 and 0.60–3.16 for *MA*, and 43.04 and 8.67–39.46 for *MI*.

The overall prevalence (*P_M_*), mean abundance (*MA*), and mean intensity (*MI*) of mites on *B. bowersi* were *P_M_* = 85.45%, *MA* = 45.67 mites/examined rat, and *MI* = 53.45 mites/infested rat, respectively. The community indexes of chiggers were higher than those of gamasid mites (Table 2). The *P_M_* of gamasid mites (*P_M_* = 78.18%) was higher than that of chiggers (*P_M_* = 52.73%) (*χ*^2^ = 6.79, *p* = 0.01 < 0.05). The *MA* and *MI* of chiggers (*MA* = 30.76, *MI* = 58.34), however, were higher than those of gamasid mites (*MA* = 14.91, *MI* = 19.07) (*MA*: Z = 3.04, *p* = 0.08 > 0.05; *MI*: Z = 2.93, *p* = 0.09 > 0.05) (Table 2). Of the five dominant mite species, *L. liui* had the highest *P_M_* (*P_M_* = 63.64%, *χ*^2^ = 79.71, *p* = 0.00 < 0.0001), *L. scutellare* had the highest *MA* (*MA* = 7.76, Z = 70.58, *p* = 0.00 < 0.0001), and *L. muntiaci* had the highest *MI* (*MI* = 355.00, Z = 11.99, *p* = 0.02 < 0.05) (Figure 4). Of the seven vector mite species found on *B. bowersi*, the *P_M_* and *MA* of *L. scutellare* (*P_M_* = 21.82%, *MA* = 7.76) were higher than those of other six vector species (*P_M_*: *χ*^2^ = 23.07, *p* = 0.00 < 0.001; *MA*: Z = 28.26, *p* = 0.00 < 0.001) (Figure 4). The difference in *MI* among seven vector species was of no statistical significance (Z = 3.99, *p* = 0.68 > 0.05).

Of the 820 individuals of gamasid mites, no larval-stage gamasid mites were collected. In terms of the sexes of gamasid mites, more females chose to parasitize the body surface of *B. bowersi* with a high sex ratio (79.40–85.80%) (Table 3), and the *MA* and *P_M_* of females (*MA* = 10.87, *P_M_* = 78.18%) were much higher than those of males (*MA*: Z = 23.42, *p* = 0.00 < 0.0001; *P_M_*: *χ*^2^ = 21.29, *p* = 0.00 < 0.0001) (Table 4). Among several life stages of gamasid mites, the number of adults was much higher than that of immature mites, including larvae (L), protonymph (N1), and deutonymph (N2). The *MA* and *P_M_* of adult gamasid mites (*MA* = 12.67, *P_M_* = 80.00%) were also higher than those of larvae, protonymph, and deutonymph (*MA*: Z = 111.69, *p* = 0.00 < 0.0001; *P_M_*: *χ*^2^ = 110.94, *p* = 0.00 < 0.0001).

Mite infestation on *B. bowersi* seemed to have a sex and age bias of the host. The infestation indexes (*P_M_*, *MA* and *MI*) of chiggers were higher on female rat hosts than on male ones (*P_M_*: *χ*^2^ = 2.23, *p* = 0.14 > 0.05; *MA*: Z = 3.53, *p* = 0.06 > 0.05; *MI*: Z = 1.71, *p* = 0.19 > 0.05) while the infestation indexes of gamasid mites were higher on male hosts than on female ones (*P_M_*: *χ*^2^ = 0.00, *p* = 1.00 > 0.05; *MA*: Z = 0.12, *p* = 0.73 > 0.05; *MI*: Z = 0.17, *p* = 0.68 > 0.05). Except for the *P_M_* of gamasid mites (*P_M_*: *χ*^2^ = 0.08, *p* = 1.00 > 0.05), most infestation indexes of mites were higher on adult hosts than on juveniles (chiggers: *P_M_*: *χ*^2^ = 1.88, *p* = 0.34 > 0.05; *MA*: Z = 2.40, *p* = 0.12 > 0.05; *MI*: Z = 0.98, *p* = 0.32 > 0.05; gamasid mites: *MA*: Z = 0.39, *p* = 0.53 > 0.05; *MI*: Z = 1.15, *p* = 0.28 > 0.05). The differences in all infestation indexes (*P_M_*, *MA* and *MI*) on different sexes and ages of hosts, however, were of no statistical significance (*p* > 0.05) (Table 5).

**Figure 5:**
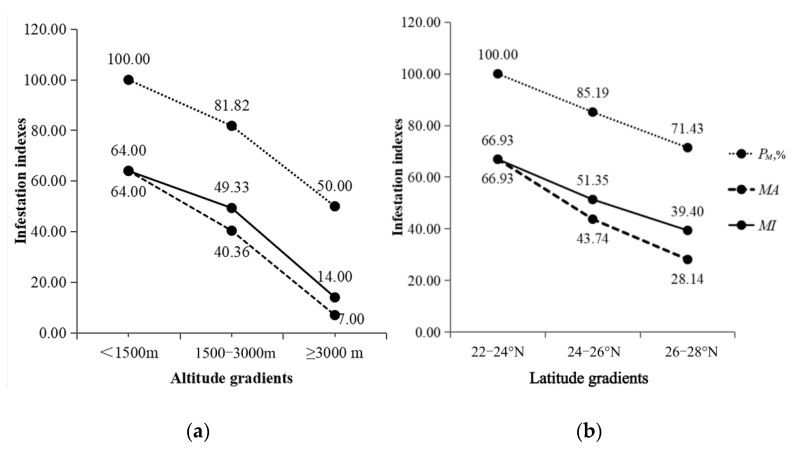
Infestation fluctuation of mites on *B. bowersi* along different altitude (**a**) and latitude (**b**) gradients in southwest China (2001–2024). Annotation: Altitude gradients (**a**): The standard deviation and confidence intervals are 78.33 and 27.58–68.36 for *MA*, and 82.33 and 32.85–78.30 for *MI*; Latitude gradients (**b**): The standard deviation and confidence intervals are 78.33 and 26.06–66.93 for *MA*, and 82.33 and 31.26–79.56 for *MI*.

The infestation indexes (*P_M_*, *MA* and *MI*) of mites on *B. bowersi* showed a fluctuation along different environmental gradients. Along different altitude gradients, the infestation indexes were higher, at < 1500 m, than at other altitude gradients (*P_M_*: *χ*^2^ = 7.46, *p* = 0.02 < 0.05; *MA*: Z = 5.74, *p* = 0.06 > 0.05; *MI*: Z = 1.18, *p* = 0.55 > 0.05). Along different latitude gradients, the infestation indexes were higher, at 22–24° N, than at other latitude gradients (*P_M_*: *χ*^2^ = 4.60, *p* = 0.04 < 0.05; *MA*: Z = 3.36, *p* = 0.19 > 0.05; *MI*: Z = 0.60, *p* = 0.74 > 0.05) (Figure 5).

### 3.3. Mutual Relationships of Mites on B. bowersi

**Figure 6:**
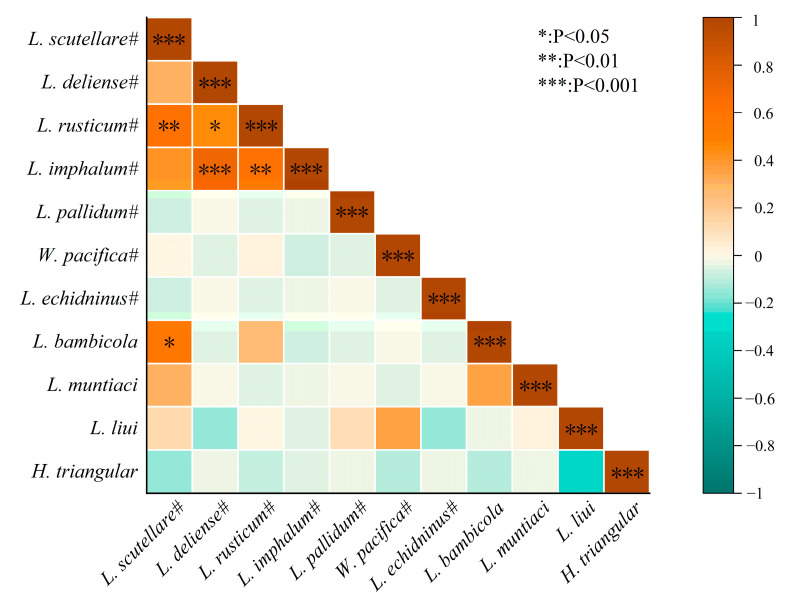
The heat map of interspecific relationships among dominant and vector species of mites on *B. bowersi* in southwest China (2001–2024). Annotation: The mite species marked with “#” are the vector species, which can serve as the vectors or potential vectors of scrub typhus and HFRS [57,58,59,60,61,62,63,64].

The association coefficient (*V*) was used to analyze the mutual relationship between two groups of mites, and the result showed that a slight positive association existed between chiggers and gamasid mites, with *V* = 0.31 (*χ*^2^ = 5.25, *p* = 0.00 < 0.05) (Table 6). Based on calculating Spearman correlation coefficients (r), the heat map was used to visualize the interspecific relationship between any 2 of 11 mite species (dominant and vector species) on *B. bowersi*. It was found that a significant positive correlation existed between five pairs of mites: *L. scutellare* and *L. rusticum* (r = 0.61), *L. scutellare* and *L. bambicola* (r = 0.58), *L. deliense* and *L. rusticum* (r = 0.46), *L. deliense* and *L. imphalum* (r = 0.71), and *L. rusticum* and *L. imphalum* (r = 0.61) (*p* < 0.05, Figure 6).

### 3.4. Estimation of the Number of Mite Species

**Figure 7:**
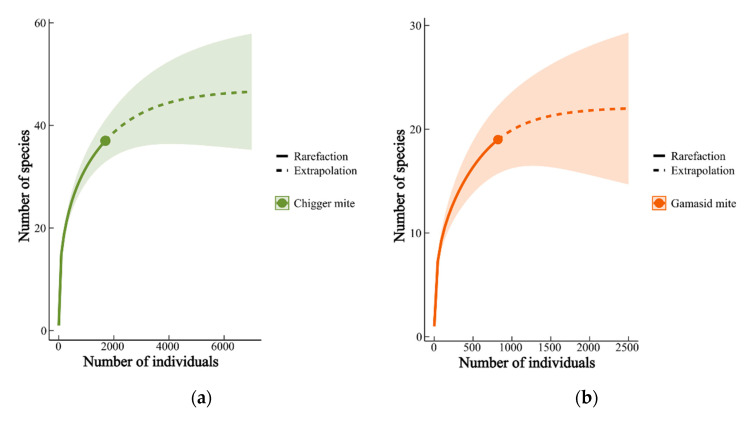
Species rarefaction and extrapolation curves of chigger mites (**a**) and gamasid mites (**b**). Annotation: The solid line in the figure is a rarefaction curve representing the observed values, while the dashed line is the extrapolation curve representing the estimated values.

Figure 7 shows the species rarefaction and extrapolation curves of chigger mites and gamasid mites, in which the shadows represent a 95% confidence interval. The increase in mites (“Number of individuals” in Figure 7) was quick in the beginning, then gradually slowed, and eventually tended to stop (Figure 7). The total number of chigger mite species was estimated to be 47 (standard error = 13) among 7000 individuals, and the total gamasid mite species were estimated as 22 (standard error = 7) among 2500 individuals.

## 4. Discussion

In the present study, there were only 55 Bower’s white-toothed rats (*B. bowersi*) captured from 10 out of 117 survey sites across the five provincial regions of southwest China (Figure 1), and the results indicate that although *B. bowersi* is distributed in southwest China, it is not one of the dominant rodent species in the region when compared with other rodent species with large populations, such as *Rattus tanezumi* and *Apodemus latronum*. The previous studies on chiggers showed that 2919 *Rattus tanezumi* rats and 501 *Apodemus latronum* mice were once captured from 56 out of 91 and 17 out of 114 survey sites in southwest China [65,66]. Although *B. bowersi* had a small population in southwest China, it harbored abundant chiggers and gamasid mites with heavy infestation burdens, and the species and infestation intensity of chiggers (37 species, *MA* = 30.76, *MI* = 58.34) were obviously higher than those of gamasid mites (19 species, *MA* = 14.91, *MI* = 19.07) (Table 1 and Table 2). The infestation indexes of chiggers on *B. bowersi* (*P_M_* = 52.73%, *MA* = 30.76, *MI* = 58.34) are much higher than those on *R. tanezumi* (*P_M_* = 21.10%, *MA* = 7.01, *MI* = 33.20) and *A. latronum* (*P_M_* = 19.76%, *MA* = 1.86, *MI* = 9.42) [65,66]. The results indicate that *B. bowersi* has a high susceptibility to infestation with mites, especially chiggers.

It has been confirmed that six chigger species are the main vectors of scrub typhus in China, and over ten chigger species are secondary or potential vectors of the disease. The six main vector species are *L. deliense*, *L. scutellare*, *L. rubellum*, *L. sialkotense* (*L. jishoum*), *L. wenense* (*L. kaohuensis* or *L. gaohuensis*), and *L. insulare* [60,62]. *Leptotrombidium scutellare* is also a potential vector of HFRS [61,67]. Among the 56 mite species identified from *B. bowersi* in the present study, there were 7 vector mite species, of which *L. scutellare* was also the most dominant species of chigger (*C_r_* = 25.24%, 427/1692). Besides *L. scutellare*, there were other vector species of mites found on *B. bowersi* (Table 1), of which *L. deliense* is one of the six main vectors of scrub typhus in China [60,62,68]. The occurrence of these vector mites (especially *L. scutellare*) will increase the potential risk of transmission and focus persistence of scrub typhus and HFRS in southwest China.

The life cycle of gamasid mites includes five stages: egg, larvae, protonymph, deutonymph, and adult (female and male) [43]. In the present study, only the adult, protonymph, and deutonymph stages of gamasid mites were found from *B. bowersi*. The number of female individuals of the two dominant species and all species of gamasid mites was higher than that of males, which is consistent with previous research results [49] (Table 3). The high sex ratio and infestation indexes of females indicate that *B. bowersi* is more susceptible to infection by female gamasid mites, possibly due to the reproductive mode of most gamasid mite species being “parthenogenesis” [43,69]. In addition, the results showed that the number and infestation indexes of adult gamasid mites were much higher than those of immature mites (Table 3 and Table 4). The immature stages of some mites have weak resistance to external environments and predators, leaving them prone to death or elimination. Additionally, the larva and nymph development periods of most gamasid mites are rapid and short. Furthermore, some gamasid mites only parasitize the host’s body surface when feeding on blood, and they will leave the host after a full meal to lay eggs in host nests, making it challenging to collect immature mites from the body surface of the host, which may explain why no larvae and only a few nymphs were collected in this study [43,69].

A sex and age bias of hosts is very common in parasite infection, including ectoparasite infestation [70,71,72]. The infestation indexes of mites varied on different sexes and ages of *B. bowersi*, which probably reflects the sex and age bias of hosts to mite infestation (Table 5). The differences in all infestation indexes (*P_M_*, *MA,* and *MI*) on different sexes and ages of hosts, however, were of no statistical significance (p > 0.05), which indicates that the above results may be unstable and unreliable, and the sampling error cannot be excluded because of small rat samples (only 55 *B. bowersi* rats captured). To confirm the sex and age bias of *B. bowersi* rats to mite infestation, more rat samples are needed in future studies.

The fluctuation of mites on *B. bowersi* along different environmental gradients (Figure 5) indicates the environmental heterogeneity of the same rodent species to mite infestation [35,65]. The higher infestation index (*P_M_*) at <1500 m suggests that *B. bowersi* may be more vulnerable to mite infestation and more easily infested with mites at low altitudes than at high altitudes.

The slight positive association between two groups of mites (chiggers and gamasid mites) suggests that chiggers and gamasid mites may have a tendency to coexist on *B. bowersi* rats, which reflects the mutual relationship between these two groups of mites. On the other hand, the positive correlation between seven pairs of mite species reflects the interspecific relationship of these mite species, which suggests that they tend to select the same individuals of their host, *B. bowersi* (Table 6) [47,50,73].

According to the relevant functions in the “INEXT” package, the estimated number of chigger species is 47, and the estimated number of gamasid mite species is 22. In this study, 37 chigger species and 19 gamasid mite species were identified, indicating that a few mite species were not collected. It is inevitable that some rare species are “missed” during field sampling investigation as some rare species are so few in number that they are difficult to collect [47,73].

The present study retrospectively analyzed the overall abundance and infestation of mites on Bower’s white-toothed rat (*B. bowersi*) in southwest China. The result revealed that *B. bowersi* had very high infestation indexes of mites with heavy mite burdens, indicating that *B. bowersi* has a very high susceptibility to mite infestation (Table 2). In the present investigation, however, only 55 *B. bowersi* rats were captured at 10 out of 117 survey sites across the five provincial regions of southwest China, indicating that *B. bowersi* is not a dominant rodent species with a large population in the region. Because of small rat samples (only 55 *B. bowersi* rats), the mite infestation differences on different sexes and ages of hosts cannot be determined and the sampling error cannot be excluded (*p* > 0.05), which is one of the limitations of the present study. Due to the limited rat samples, this study did not explore the variability of mite abundance and infestation on *B. bowersi* in different survey sites, geographical regions, habitats, and seasons (different periods of the year), which is another limitation of this study. In order to collect more *B. bowersi* rats (more rat samples), more field investigations are needed in the future. The result of this study also showed that the mite infestation indexes on *B. bowersi* were high at the low-latitude and -altitude gradients (Figure 5). Considering that *B. bowersi* is not a dominant rodent species in southwest China, it is recommended that field investigations should be expanded into areas with low latitudes and altitudes to collect more *B. bowersi* rats and significantly increase the sample size of the rats. Given the extremely high susceptibility of *B. bowersi* to mite infestation, it is also necessary to perform more laboratory work on *B. bowersi* and its associated mites in future studies, including the pathogen detection of related zoonoses from rat and mites.

## Figures and Tables

**Table 1:** Taxonomic identification of mites on *B. bowersi* in the five provincial regions of southwest China (2001–2024).

Taxonomic Taxa of Mites	Identified Species and Individuals of Mites (Figures in Brackets Are Corresponding Individuals)
Trombiculidae	A total of 1692 individuals in 37 species and 7 genera.
*Leptotrombidium*	*L. scutellare* (427) *; *L. sinicum* (35); *L. eothenomydis* (3); *L. hiemalis* (1); *L. rusticum* (58) *; *L. wangi* (7); *L. densipunctatum* (1); *L. yongshengense* (4); *L. deliense* (4) *; *L. xiaguanense* (35); *L. imphalum* (12) *; *L. dianchi* (4); *L. jinmai* (1); *L. allosetum* (2); *L. pallidum* (1) *; *L. hsui* (11); *L. longchuanense* (38); *L. qiui* (1); *L. suense* (51); *L. baoshui* (107); *L. bambicola* (266); *L. muntiaci* (355).
*Trombiculindus*	*T. cuneatus* (1); *T. bambusoides* (1); *T. nujiange* (2).
*Neotrombicula*	*N. japonica* (2); *N. vulgaris* (5).
*Doloisia*	*D. manipurensis* (2).
*Ascoschoengastia*	*A. yunwui* (1).
*Herpetacarus*	*H. aristoclavus* (3); *H. fukienensis* (7).
*Walchia*	*W. pacifica* (97) *; *W. parapacifica* (1); *W. micropelta* (11); *W. kor* (2); *W. nanfangis* (1); *W. sheensis* (132).
Laelapidae	A total of 814 individuals in 17 species and 6 genera.
*Laelaps*	*L. echidninus* (27) *; *L. guizhouensis* (2); *L. paucisetosa* (1); *L. turkestanicus* (14); *L. traubi* (2); *L. liui* (416); *L. taingueni* (1).
*Haemolaelaps*	*H. triangular* (221); *H. traubi* (3); *H. petauristae* (1); *H. anomalis* (1).
*Dipolaelaps*	*D. jiangkouensis* (55); *D. anourosorecis* (16).
*Hypoaspis*	*H. pavlovskii* (44); *H. miles* (1).
*Androlaelaps*	*A. singularis* (2).
*Hirstionyssus*	*H. sunci* (7).
Blattisocidae	A total of 4 individuals in 1 species and 1 genus.
*Proctolaelaps*	*P. pygmaeus* (4).
Aceosejidae	A total of 2 individuals in 1 species and 1 genus.
*Lasioseius*	*L. medius* (2).

Annotation: Species marked with “*” are the vector species, which can be the vectors or potential vectors of scrub typhus and HFRS [57,58,59,60,61,62,63,64].

**Table 2:** Community and infestation indexes of mites on *B. bowersi* in southwest China (2001–2024).

Taxa of Mites	Community Indexes				Infestation Indexes		
	*H′*	*D*	*M_f_*	*E*	*P_M_*, %	*MA*	*MI*
Chiggers	2.28	0.85	4.84	0.63	52.73	30.76	58.34
Gamasid mites	1.48	0.66	2.68	0.50	78.18	14.91	19.07
Total	2.65	0.90	7.03	0.66	85.45	45.67	53.45

Annotation: The standard deviation and confidence intervals are 56.20 and 13.85–34.87 for *MA*, and 65.83 and 20.20–51.01 for *MI*.

**Table 3:** The sex ratio and age structure of two dominant gamasid mite species and all gamasid mites on *B. bowersi* in southwest China (2001–2024).

Two Dominant and All Gamasid Mites	Number and Sex Ratio of Females		Number and Sex Ratio of Males		Adult	L	N1	N2	Immature
	No.	Sex Ratio, %	No.	Sex Ratio, %	*C_r_*, %	No.	No.	No.	*C_r_*, %
*L. liui*	289	79.40	75	20.60	87.50	0	8	44	12.50
*H. triangular*	145	91.77	13	8.23	71.49	0	0	63	28.51
All gamasid mites	598	85.80	99	14.20	85.00	0	10	113	15.00

Annotation: L = larvae, N1 = protonymph, and N2 = deutonymph.

**Table 4:** Infestation indexes of different sexes and ages of gamasid mites on the body surface of *B. bowersi* in southwest China (2001–2024).

Sexes and Ages of Gamasid Mites		Infected Host	*P_M_*, %	*MA*	*MI*
Sexes	Female	43	78.18	10.87	13.91
	Male	19	34.55	1.80	5.21
Ages	Adult	44	80.00	12.67	15.84
	Larva	0	/	0.00	/
	Protonymph	2	3.64	0.18	5.00
	Deutonymph	16	29.09	2.05	7.06

Annotation: Sexes: The standard deviations and confidence intervals are 15.94 and 3.65–9.60 for *MA*, and 19.95 and 6.82–16.66 for *MI*. Ages: The standard deviations and confidence intervals are 13.41 and 2.11–5.71 for *MA*, and 22.76 and 8.31–19.79 for *MI*. The “/” symbol indicates cases where the calculation was not possible due to a denominator of zero.

**Table 5:** Infestation indexes of mites on different sexes and ages of *B. bowersi* in southwest China (2001–2024).

Sexes and Ages of *B. bowersi*	No. of *B. bowersi*	Infestation Indexes of Chiggers			Infestation Indexes of Gamasid Mites		
		*P_M_*, %	*MA*	*MI*	*P_M_*, %	*MA*	*MI*
Females	27	62.96	49.67	78.88	81.48	11.15	13.68
Males	28	42.86	12.54	29.25	82.14	18.54	22.57
Total	55	52.73	30.76	58.34	81.82	14.91	18.22
Adults	48	56.25	34.73	61.74	81.25	15.98	19.67
Juveniles	7	28.57	3.57	12.50	85.71	7.57	8.83
Total	55	52.73	30.76	58.34	81.82	14.91	18.22

Annotation: Sexes: Chiggers: The standard deviation and confidence intervals are 73.88 and 13.78–51.97 for *MA*, and 94.13 and 28.04–94.93 for *MI*; Gamasid mites: The standard deviation and confidence intervals are 28.07 and 8.20–23.45 for *MA*, and 30.08 and 10.71–27.98 for *MI*. Ages: Chiggers: The standard deviation and confidence intervals are 78.33 and 13.93–50.32 for *MA*, and 94.13 and 26.73–97.54 for *MI*; Gamasid mites: The standard deviation and confidence intervals are 28.07 and 8.20–23.45 for *MA*, and 30.08 and 10.89–28.54 for *MI*.

**Table 6:** Analysis of the mutual relationship between two groups of mites (chiggers and gamasid mites) on *B. bowersi* hosts in southwest China (2001–2024).

Mutual Relationship Between Two Groups of Mites		Gamasid Mites		Total
		No. of Infested Hosts (+)	No. of Uninfested Hosts (−)	
Chiggers	No. of infested hosts (+)	27	2	29
	No. of uninfested hosts (−)	18	8	26
Total		45	10	55
Association coefficient	*V* = 0.31			
Chi-square	*χ*^2^ = 5.25			
Significance	*p* = 0.00 < 0.001			

## Data Availability

The experimental data used to support the findings of this study are available from the corresponding author on request.
